# Can Falls Risk Prediction Tools Correctly Identify Fall-Prone Elderly Rehabilitation Inpatients? A Systematic Review and Meta-Analysis

**DOI:** 10.1371/journal.pone.0041061

**Published:** 2012-07-17

**Authors:** Bruno Roza da Costa, Anne Wilhelmina Saskia Rutjes, Angelico Mendy, Rosalie Freund-Heritage, Edgar Ramos Vieira

**Affiliations:** 1 Division of Clinical Epidemiology and Biostatistics, Institute of Social and Preventive Medicine, University of Bern, Bern, Switzerland; 2 Department of Epidemiology and Biostatistics, Robert Stempel School of Public Health, Florida International University, Miami, Florida, United States of America; 3 Glenrose Rehabilitation Hospital, Edmonton, Alberta, Canada; 4 Department of Physical Therapy, Florida International University, Miami, Florida, United States of America; Tehran University of Medical Sciences, Islamic Republic of Iran

## Abstract

**Background:**

Falls of elderly people may cause permanent disability or death. Particularly susceptible are elderly patients in rehabilitation hospitals. We systematically reviewed the literature to identify falls prediction tools available for assessing elderly inpatients in rehabilitation hospitals.

**Methods and Findings:**

We searched six electronic databases using comprehensive search strategies developed for each database. Estimates of sensitivity and specificity were plotted in ROC space graphs and pooled across studies. Our search identified three studies which assessed the prediction properties of falls prediction tools in a total of 754 elderly inpatients in rehabilitation hospitals. Only the STRATIFY tool was assessed in all three studies; the other identified tools (PJC-FRAT and DOWNTON) were assessed by a single study. For a STRATIFY cut-score of two, pooled sensitivity was 73% (95%CI 63 to 81%) and pooled specificity was 42% (95%CI 34 to 51%). An indirect comparison of the tools across studies indicated that the DOWNTON tool has the highest sensitivity (92%), while the PJC-FRAT offers the best balance between sensitivity and specificity (73% and 75%, respectively). All studies presented major methodological limitations.

**Conclusions:**

We did not identify any tool which had an optimal balance between sensitivity and specificity, or which were clearly better than a simple clinical judgment of risk of falling. The limited number of identified studies with major methodological limitations impairs sound conclusions on the usefulness of falls risk prediction tools in geriatric rehabilitation hospitals.

## Introduction

Patient falls is a predominant patient safety issue in hospitals accounting for up to 32.3% of all reported patient safety incidents [1]. Fall-related complications lead to a prolonged rehabilitation period and increased health care costs [2], [3]. It is estimated that just in the United Kingdom, patient falls in acute care hospitals cost approximately 92 million pounds per year [4]. The actual costs of inpatient falls may be even higher as falls are frequently underreported [1]. Other than the cost of falls to hospitals, patients incur additional costs as 35% of the patients who fall suffer physical harm or even death [1]. Falls may also cause fear of falling, which may lead to immobility and its complications such as muscle weakness, contracture, postural hypotension, and thrombogenic events [5], [6].

Falls are the first leading cause of unintentional injury-related death among the elderly (i.e. people 65 years and older) [7]. Falls cause more than 95% of all hip fractures in the elderly; 20% of the elderly people who suffer hip fractures die within a year [8]. The prevalence rate of falls in acute hospitals is around two to six percent, [9] in general rehabilitation settings is 12.5%, [3], [10] and in geriatric rehabilitation hospitals is 24 to 30% [11], [12]. The higher prevalence of falls in geriatric rehabilitation hospitals may be explained by the fact that elderly patients are generally frailer, are more exposed to risk factors for falling than younger patients, and are encouraged in rehabilitation settings to be physically active, independent, and involved in rehabilitation activities [3], [13]. These circumstances challenge their physical abilities, and places them in situations where they are more likely to fall [3]. Thus, elderly patients in rehabilitation hospitals are particularly at risk for falls.

Although there is a clear need to implement strategies to prevent elderly inpatient falls in rehabilitation hospitals, it is unclear which strategies are the most effective for fall prevention in this population [14]. A common strategy is the use of falls risk prediction tools [4]. Identifying fall-prone patients on admission may help prevent falls by guiding implementation of targeted fall prevention strategies. However, the accuracy of the available prediction tools in actually identifying fall-prone patients is debated [15], [16]. Using inaccurate falls prediction tools may create a false sense of safety on both patients and staff, leaving patients at risk exposed to the potential adverse effects of falling and consequent injuries [15]. It is not clear at the moment if there is an efficient tool to assess the risk of falls among rehabilitation hospital elderly inpatients. Therefore, the objective of this study was to systematically review the literature to identify the falls prediction tools available for assessing elderly inpatients in rehabilitation hospitals, and to assess the prediction usefulness of these tools.

## Methods

### Literature Search

To identify eligible studies we undertook a systematic search of 6 databases (MEDLINE, CINAHL, SCOPUS, Web of Science, Rehab data, and CIRRIE Database of International Rehabilitation Research). The search strategy used a combination of terms for rehabilitation hospital inpatient, falls, risk assessment, prediction, and older age. The terms included text words, keywords and subject headings specific to each database (Appendix S1). Similar strategies were used to identify previously published systematic reviews in three databases (Cochrane Database of Systematic Reviews, OTseeker, and PEDro). To try and minimize the chance of publication bias, we conducted a thorough search of unpublished studies. We searched ProQuest Dissertations for unpublished studies and searched conference proceedings on OCLC ProceedingsFirst. We also screened reference lists of included papers and contacted authors and experts in the field. All searches were conducted from databases inception to July 2011. Our systematic review has no published protocol available.

### Study Selection and Outcomes of Interest

To be included in our review, studies must have conducted a prospective investigation of the predictive properties of prediction tools for falls of elderly (i.e. ≥65 years of age) inpatients in rehabilitation hospitals. Only studies published in the English language were considered for inclusion. In addition, studies should have either reported our primary outcome of interest with respective confidence intervals (i.e. sensitivity and specificity of prediction tools of falls among elderly rehabilitation inpatients) or have reported enough data so that we could construct 2×2 tables and directly calculate these estimates. Positive and predictive values were secondary outcomes of interest, and were also extracted whenever available. Two reviewers (BRDC, ERV) independently screened the titles and abstracts of all identified citations and subsequently assessed full text versions of potentially eligible studies for inclusion. Disagreements regarding study eligibility were resolved through discussion.

### Data Collection

Two reviewers (BRDC, ERV) trained in health research methodology extracted data independently and in duplicate using a standardized form. Data regarding participants’ characteristics, prediction tools used, main findings, and methodological quality were extracted and tabulated. Disagreements regarding extracted data were resolved through discussion.

### Methodological Quality Assessment

We assessed the following study characteristics deemed important for the development of risk prediction tools: [17], [18] (1) Fall or faller clearly defined: Was a clear definition of the outcome “fall” or “faller” explained and standardized among staff? (e.g. an incident in which a patient suddenly and involuntarily came to rest upon the ground or surface lower than their original station) [19]; (2) Blinded adjudication of event: Were staff responsible for counting falls/identify fallers blinded to the estimates produced by the prediction tool?; (3) Confounding assessed: Were other relevant patient characteristics taken into account when interpreting results? (i.e. difference between groups regarding relevant risk factors not covered by the predicting tool); (4) Cut-score pre-defined: If a single cut-score was used to report estimates, was it based on previous evidence and defined *a priori*?; (5) Prediction tool compared to clinical judgment: Was the prediction tool compared to staff’s intuitive estimates (best guess)?

### Statistical Analysis

Description of the characteristics of the included studies were tabulated and presented in terms of absolute and relative frequencies, sensitivities and specificities, negative- and positive predictive values and corresponding 95% confidence intervals. We illustrated the data by plotting sensitivities and specificities in ROC space graphs, which allows the visual inspection of between-study heterogeneity. For meta-analytical purposes, we pre-specified to summarize the data applying the cut-scores that were either considered standard or were reported to optimally balance sensitivity against specificity. Only the STRATIFY tool had enough data to be meta-analyzed in the present investigation. It ranges from zero to five and the cut-score of ≥2 was considered for meta-analysis [20]. We meta-analyzed sensitivities and specificities using the ‘metandi’ module in STATA (version 11.2) [21]. To perform a meta-analysis of sensitivities and specificities with three studies, we used a univariate version of ‘metandi’, which was kindly provided to us by the University of Bristol.

## Results

**Figure 1:**
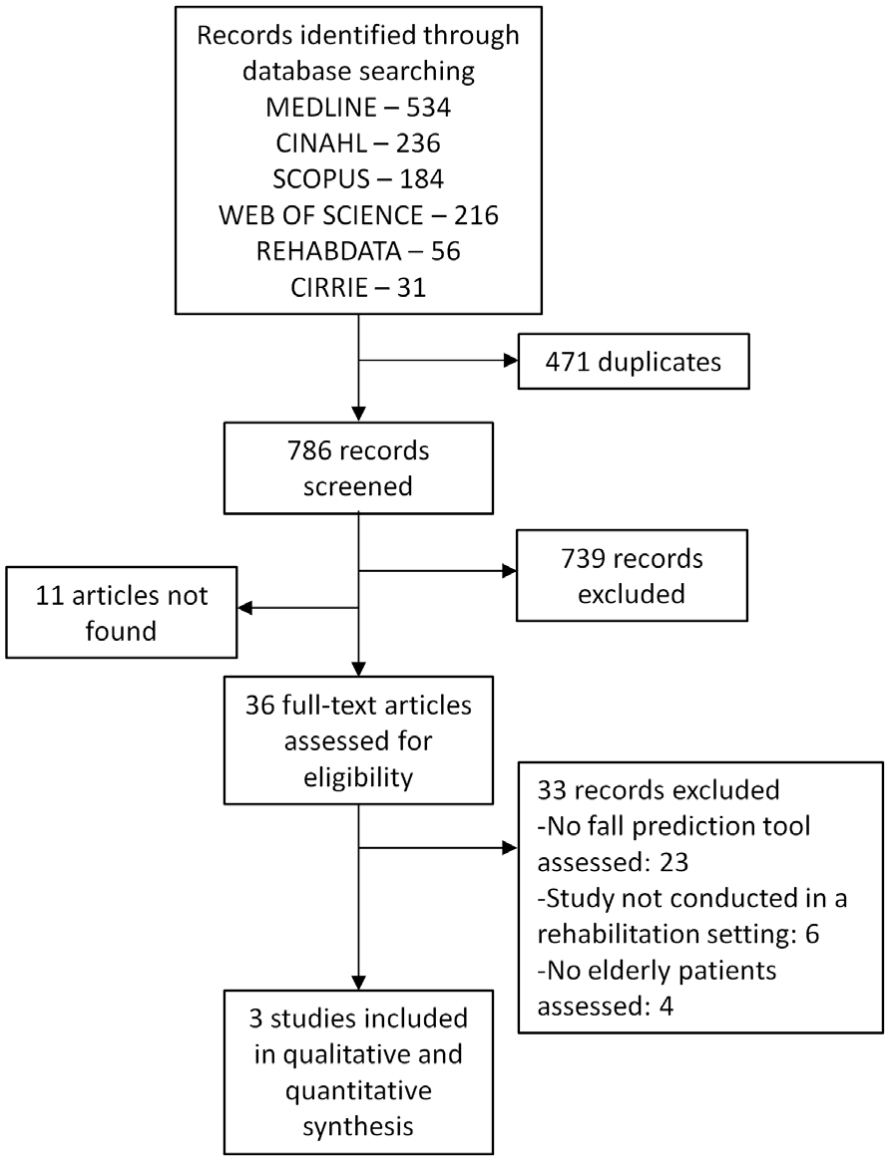
Flow-diagram depicting the selection process of studies investigating risk assessment tools for elderly inpatient falls in rehabilitation hospitals.

We identified 1257 references in our literature search and considered 786 to be potentially eligible (Figure 1). After full text screening, three studies met our inclusion criteria.

### Description of the Included Studies

Overall, three studies including 754 elderly inpatients in rehabilitation wards/hospitals were identified by our search strategy (Table 1). The median year of publication was 2006 (range, 2003 to 2008). The average age of the patients ranged from 79 to 81 years, the percentage of female subjects ranged from 62 to 69%, and the proportion of fallers ranged from 26 to 51%. Cooker & Oliver did not report the number of fallers in their study. All included studies used a prospective cohort design. Two studies reported diagnosis of study participants which consisted mostly of orthopedic and neurological conditions [20], [22]. Fall rates per 1000 patient-days were 13.4 in the study of Cooker & Oliver and 14.7 in the study of Haines et al [20], [22]. Vassallo et al. did not report length of follow-up [23].

**Table 1:** Overview of included studies showing studies characteristics and summary of findings.

Ref.	Sampling	n*	Mean Age	%♀	Fall data source	#falls	Fallers (%)	Reported findings
Cooker 2003	Patients admitted to a Geriatric Assessment and Rehabilitation Unit. Mean length of patient stay was 50 days.	432	81	69	Patient incident report	13.4 falls/1000 patient days	–	**STRATIFY (cut-off score ≥1)**
								sensitivity: 95% (95%CI 90–99)
								specificity: 17% (95%CI 13–21)
								positive predictive value: 28% (95%CI 24–33)
								negative predictive value: 90% (95%CI 83–98)
								**STRATIFY (cut-off score ≥2)**
								sensitivity: 66% (95%CI 57–75)
								specificity: 47% (95%CI 41–52)
								positive predictive value: 30% (95%CI 24–36)
								negative predictive value: 80% (95%CI 74–85)
								**STRATIFY (cut-off score ≥3)**
								sensitivity: 36% (95%CI 27–45)
								specificity: 85% (95%CI 81–89)
								positive predictive value: 45% (95%CI 35–55)
								negative predictive value: 79% (95%CI 75–84)
								**STRATIFY (cut-off score ≥4)**
								sensitivity: 11% (95%CI 5–17)
								specificity: 96% (95%CI 94–98)
								positive predictive value: 50% (95%CI 30–70)
								negative predictive value: 76% (95%CI 72–80)
								**STRATIFY (cut-off score = 5)**
								sensitivity: 9% (95%CI −0.9–3)
								specificity: 100% (95%CI 99–100)
								positive predictive value: 50% (95%CI –19–119)
								negative predictive value: 74% (95%CI 70–79)
Haines 2006	Patients consecutively admitted at a hospital. metropolitan rehabilitation and aged care Rate of falls per 1000 patient-days was reported but exact length of follow-up is unclear.	122	79	69	Patient incident report	14.7 falls/1000 patient days	26	**STRATIFY (cut-off score ≥1)**
								sensitivity: 96% (95%CI 86–100)
								specificity: 20% (95%CI 12–29)
								**STRATIFY (cut-off score ≥2)**
								sensitivity: 77% (95%CI 59–92)
								specificity: 51% (95%CI 41–61)
								**STRATIFY (cut-off score ≥3)**
								sensitivity: 42% (95%CI 24–63)
								specificity: 78% (95%CI 70–86)
								**STRATIFY (cut-off score ≥4)**
								sensitivity: 4% (95%CI 0–14)
								specificity: 93% (95%CI 88–98)
								**PJC-FRAT (Falls risk alert card)**
								sensitivity: 73% (95%CI 55–90)
								specificity: 75% (95%CI 66–83)
								**PJC-FRAT (Exercise program)**
								sensitivity: 12% (95%CI 3–27)
								specificity: 84% (95%CI 77–91)
								**PJC-FRAT (Education program)**
								sensitivity: 27% (95%CI 12–46)
								specificity: 68% (95%CI 58–77)
								**PJC-FRAT (Hip protectors)**
								sensitivity: 31% (95%CI 14–48)
								specificity: 90% (95%CI 83–95)
Vassallo 2008	Consecutive patients from rehabilitation ward of a rehabilitation hospital admitting elderly patients. Length of follow-up unclear.	200	81	62	Falls diary compiled by nurses	–	51 (length of follow-up unclear)	**STRATIFY (cut-off score ≥2)**
								sensitivity: 82% (95%CI 69–90)
								specificity: 34% (95%CI 27–42)
								positive predictive value: 30% (95%CI 23–38)
								negative predictive value: 85% (95%CI 73–91)
								**DOWNTON (score ≥3)**
								sensitivity: 92% (95%CI 82–97)
								specificity: 36% (95%CI 28–43)
								positive predictive value: 33% (95%CI 25–41)
								negative predictive value: 93% (95%CI 83–97)
								**Clinical judgment (observation of wandering behavior)**
								sensitivity: 43% (95%CI 30–56)
								specificity: 91% (95%CI 84–94)
								positive predictive value: 61% (95%CI 44–75)
								negative predictive value: 82% (95%CI 75–87)

* Number of patients;

♀ Number of females.

### Quality Assessment

The methodological limitations of the studies are presented on Table 2. In two out of three studies adjudicators were unblinded or it was unclear whether adjudicators were blinded to the baseline score of the predicting tools which was established at study entry. One out of three studies did not report whether a “fall” definition was pre-established. Two out of three studies did not compare the performance of the prediction tool to staff’s intuitive estimates (best guess).

**Table 2:** Assessment of potential threats to internal/external validity of included studies.

Study	Fall or faller clearly defined	Blinded adjudicationof event	Confounding assessed	Cut-scorepre-defined	Prediction tool compared to clinical judgment^*^
**Coker 2003**					
STRATIFY	+	?	−	NA	−
**Haines 2006**					
STRATIFY	+	+	+	NA	−
PJC-FRAT	+	−	+	NA	−
**Vassallo 2008**					
STRATIFY	?	?	−	+	+
DOWNTON	?	?	−	+	+
Clinical	?	?	−	+	NA
Judgement					

+: the criterion was satisfied; –: the criterion was not satisfied; ?: it was unclear whether the criterion was satisfied; NA: Not applicable; ^*^comparison of sensitivity.

### Fall Prediction Tools

All three studies investigated the predictive properties of the STRATIFY tool. Two of the studies also used other fall prediction tools: Haines et al. also used the PJC-FRAT, and Vassallo et al. also used the DOWNTON Fall Risk Index and “clinical judgment” [22], [23].

### Estimates

Table 1 displays results extracted from the three studies. In general, Haines et al. reported higher sensitivity but lower specificity of the STRATIFY tool compared to the PJC-FRAT [22]. Vassallo et al. examined the STRATIFY tool, the DOWNTON Fall Risk Index, and clinical judgment and reported that the DOWNTON Fall Risk Index showed the highest sensitivity and clinical judgment the highest specificity [23]. Cooker & Oliver examined exclusively the STRATIFY tool, and reported similar estimates of sensitivity and specificity reported by Haines et al., but somewhat different estimates than those reported by Vassallo et al [20].

**Figure 2:**
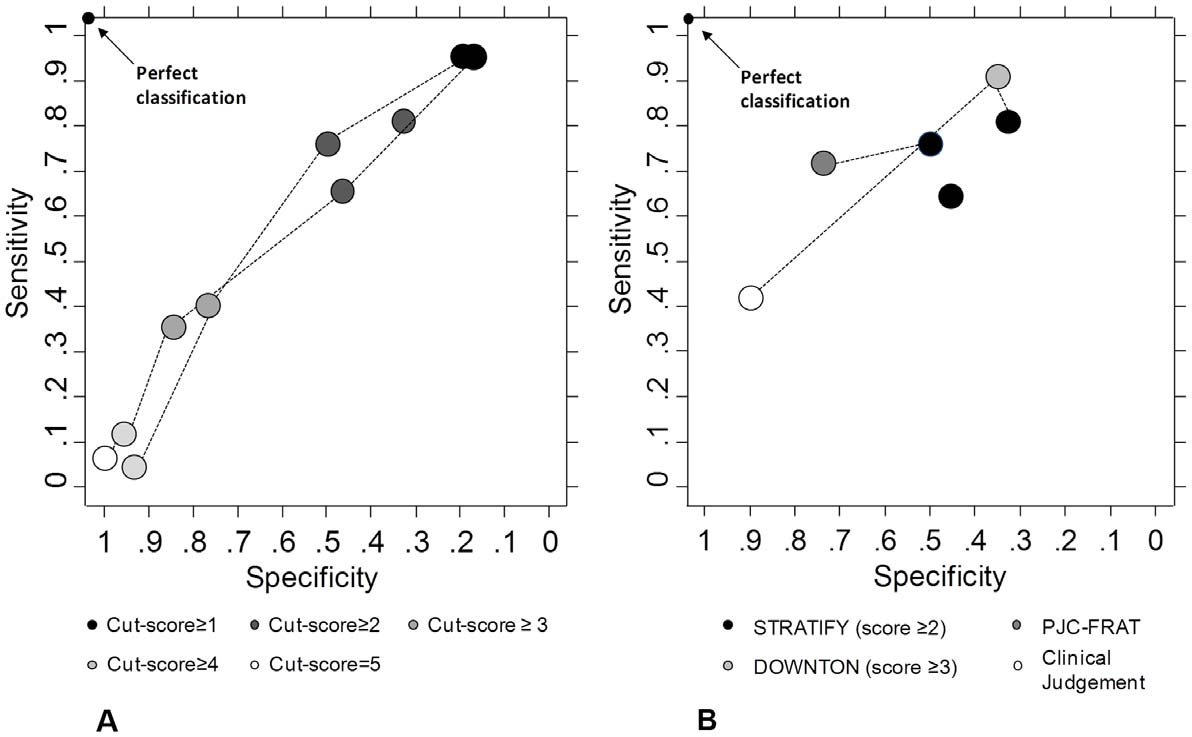
ROC space showing sensitivity and specificity of the STRATIFY tool per study for different cut-scores (**A**)**, and for fall prediction based on clinical judgment and on the optimal cut-off score of STRATIFY, DOWNTON and PJC-FRAT** (**B**)**.** Estimates originated from the same studies are connected with dashed lines. Estimates closer to the top left corner have better sensitivity-specificity.

Cooker & Oliver and Haines et al. reported estimates of sensitivity and specificity for different cut-scores of the STRATIFY tool, whereas Vassalo et al. reported these estimates only for a cut-score of two or more points (figure 2). Figure 2(A) displays sensitivity and specificity for different cut-scores of the STRATIFY tool. The closer estimates are to the top left corner, the better are their sensitivity-specificity. All three studies reported sensitivity and specificity for the STRATIFY cut-score ≥2 which allowed pooling of these estimates. Pooled sensitivity across the three studies was 73% (95%CI 63 to 81%) and pooled specificity was 42% (95%CI 34 to 51%). Visual inspection of figure 2(A) indicates moderate between-study heterogeneity in estimates. Figure 2(B) displays estimates of sensitivity and specificity for each prediction tool according to cut-scores defined by developers of these tools as their optimal cut-score. It can be seen from this graph that the DOWNTON tool has the highest sensitivity (92%), while the PJC-FRAT offers a good balance between sensitivity and specificity (73% and 75%, respectively).

## Discussion

The present systematic review identified three studies that investigated the prediction properties of different prediction tools for falls of elderly inpatients in rehabilitation hospitals: the STRATIFY, the DOWNTON, and the PJC-FRAT. The combined estimates for the three studies at the optimal cut-score of the STRATIFY tool (score ≥2) indicated that this tool has less than optimal sensitivity and specificity when applied to a population of elderly rehabilitation inpatients. The paucity in data did not allow meta-analysis of either the PJC-FRAT or DOWNTON tool. The STRATIFY gives a score which can range from zero to five, and its authors reported that a cut-score of ≥2 offers the best combination of sensitivity and specificity [20]. The PJC-FRAT is composed of four elements (falls risk alert card, additional exercise program, education program, hip protectors); the element “falls risk alert card”, which yields a simple dichotomous score “high risk of fall” or “low risk of fall”, was reported by its authors to have the best combination sensitivity-specificity [22]. The DOWNTON score can range from zero to eleven, and a cut-score of ≥3 has been determined to result in the best balance between sensitivity and specificity [23].

Two of the included studies reported sensitivity and specificity for multiple cut-scores of the STRATIFY [20], [22]. It can be seen from figure 2(A) that also in an elderly rehabilitation setting a cut-score ≥2 results in the best combination of sensitivity and specificity for this particular tool. Two studies reported sensitivity and specificity for more than one prediction tool, allowing the direct comparison of their performance to identify patients with high risk of falling. This comparative design is optimal to draw conclusions regarding which tool performs best for the identification of patients at high risk of falling. Haines et al. compared the prediction performance of the STRATIFY (cut-score ≥2) and of the PJC-FRAT (falls risk alert card) in the same patients, and reported similar values of sensitivity and specificity for both tools (figure 2(B)) [22]. Vassalo et al. also used a comparative design to assess the prediction properties of the STRATIFY (cut-score ≥2) and of the DOWNTON (cut-score ≥3) and reported that these tools had similar values of specificity but that the DOWNTON had a better sensitivity (figure 2(B)) [23]. As shown in figure 2(B), the indirect comparison of sensitivity and specificity between the falls prediction tools across all studies indicate that no single tool clearly stands out from the others as the optimal prediction tool. When identifying patients at high risk of falling, the trade-off between sensitivity and specificity is optimal when the tool correctly discriminates patients at high risk of falling from those at low risk. If we assume that sensitivity should be at least 80% to be clinically relevant when predicting fall risk, we observe that the corresponding specificity is very low, leading to many falsely labeled persons at high risk of falling which unnecessarily burdens patients and staff. It is important to stress that comparison across studies of estimates shown in figure 2(B) is indirect in nature and therefore may be misleading and must be interpreted with caution.

We observed some variation between estimates of the same tool and cut-score across studies which must also be considered when interpreting our findings. Previous reviews have linked such variation to methodological and clinical heterogeneity. A systematic review of fall prediction tools identified 35 studies conducted in acute care settings [24]. The authors reported great variation between the studies and concluded that different settings, populations, and study designs (retrospective or prospective) were responsible for the reported variation. Oliver et al. (2008) conducted a systematic review to identify all studies that had prospectively investigated the predictive property of the STRATIFY tool [17]. They identified 8 studies that reported considerably different results regarding the predictive properties of the tool. The authors also associated such variation to different settings and populations between studies. Our results show that results can vary between studies even in a similar population, setting and design. In fact, creators of the STRATIFY tool themselves have contested the usefulness of such tools claiming that it may be much better to address reversible risk factors to try and avoid patients from falling, which is supported by others [17] [23]. Oliver advocates the identification and modification of risk factors as the optimal strategy to prevent falls as opposed to “risk prediction, which may be inaccurate and does not of itself do anything to stop patients falling” [15]. Nonetheless, other creators of well known fall-risk prediction tools defend their use [16].

This is the first review to search for studies investigating the predictive properties of different fall prediction tools in an elderly population in a rehabilitation hospital setting. Our findings reveal the scarcity of effective falls risk prediction tools for this specific population which may be particularly at risk. We found only one tool (PJC-FRAT) that was developed and tested in an elderly population of a rehabilitation hospital [22]. Moreover, implementation of such tools in the clinical setting is time and money consuming and to be worth the process, they must be at least significantly better than clinicians’ clinical judgment (best guess). Vassalo et al. reported that the STRATIFY and DOWNTON had better sensitivity (82% and 92%, respectively) than clinical judgment (43%), and that both had worse specificity (34% and 36%, respectively) than clinical judgment (91%), which makes the usefulness of the these two falls prediction tools questionable [23].

Strengths of our review include an extensive search of six general and field-specific databases with a sensitive search strategy and thorough assessment of methodological quality of included studies. The major limitation of our study concern the low number of studies included. Although not a limitation which concerns the design of our review, the limited number of identified studies impairs sound conclusions to be made at this point concerning usefulness of falls risk prediction tools in geriatric rehabilitation hospitals. Moreover, we only included studies published in the English language, which have been reported to have different results than studies published in other languages [25]. However, the evidence for this potential bias is based only on studies of therapeutic interventions. Because there is currently no study which investigated whether this bias exists in systematic reviews of screening intervention studies, we do not know whether this language restriction may be indeed a potential threat to the validity of our findings [26].

Future studies with the purpose of developing new falls prediction tools should follow the rigorous steps required for such a purpose, taking into consideration the methodological issues discussed in the present review, and including suggestions for interventions rather than simply classifying the level of falls risk. In addition, future studies using prediction tools in falls prevention programs should investigate whether prediction tools are better than either simply addressing reversible risk factors or clinical judgment.

## Supporting Information

Appendix S1 **Search strategies used to identify relevant articles in each of the databases.**
(DOCX)
