# Supplementary material for: Can Falls Risk Prediction Tools Correctly Identify Fall-Prone Elderly Rehabilitation Inpatients? A Systematic Review and Meta-Analysis
Source: PLoS One. 2012 Jul 17;7(7):e41061. doi: 10.1371/journal.pone.0041061 (PMC3398864; doi:10.1371/journal.pone.0041061)
Supplement: Appendix S1 — Search strategies used to identify relevant articles in each of the databases. (DOCX) [file pone.0041061.s001.docx]

**Appendix S1.** Search strategies used to identify relevant articles in each of the databases.

**I. MEDLINE**

**Search terms for “fall”**

**1.** exp Accidental Falls/

**2.** Fall$.tw

**3.** 1 or 2

**Search terms for “rehabilitation hospital inpatient”**

**4.** Hospital$.mp

**5.** Inpatient rehabilitation.tw

**6.** 4 or 5

**Search terms for “elderly”**

**7.** exp Aged/

**8.** (senior$ or elderly or older).tw

**9.** 7 or 8

**Search term for “risk assessment”**

**10.** exp risk assessment/

**11.** (risk$ adj3 assessment$).tw

**12.** predict$.tw

**13.** 10 or 11 or 12

**Combination of search terms:**

**14.** 3 and 6 and 9 and 13

**Search terms for limiting results**

**15.** limit 14 to english language

**16.** (child$ not adult$).mp

**17.** (animal not human).mp

**18.** review.pt

**19.** 16 or 17 or 18

**20.** 15 not 19

**II. CINAHL**

**Search terms for “fall”**

**1.** (MW "Accidental Falls") or fall

**Search terms for “rehabilitation hospital inpatient”**

**2.** (MW “Rehabilitation Centers”) or (MW "Inpatients") or hospital

**Search terms for “elderly”**

**3.** ( (MW "Aged, 80 and Over") ) or Aged, 65+ years or ( senior* or elderly or older )

**Search term for “risk assessment”**

**4.** (MM "Fall Risk Assessment Tool") or TX Fall Risk Assessment Tool or (MM "Risk Assessment") or TX predict*

**Combination of search terms:**

**5.** 1 AND 2 AND 3 AND 4

**Limits provided by database**

Language: ENGLISH

**III. SCOPUS**

**Search terms for “fall”**

**1.** TITLE-ABS-KEY(fall*) AND DOCTYPE(ar OR cp)

**Search terms for “rehabilitation hospital inpatient”**

**2.** (TITLE-ABS-KEY((rehabilitation PRE/15 hospital*)) OR TITLE-ABS-KEY((rehabilitation PRE/15 inpatient*))) AND DOCTYPE(ar OR cp)

**Search terms for “elderly”**

**3.** TITLE-ABS-KEY(elderly OR older OR aged OR senior*)

**Combination of search terms:**

**4.** 1 AND 2 AND 3

**IV. WEB OF SCIENCE**

**Search terms for “fall”**

**1.** Topic=(fall*)

**Search terms for “rehabilitation hospital inpatient”**

**2.**Topic=(hospital*) AND Topic=(inpatient*)

**Search terms for “elderly”**

**3.** Topic=(elderly OR older OR aged OR senior*)

**Combination of search terms:**

**4.** #1 AND #2 AND #3

**Limits provided by database**

Language: ENGLISH

**V. REHABDATA**

**Find results with all of the words**

fall* hospital* rehabilitation

**VI. CIRRIE DATABASE OF INTERNATIONAL REHABILITATION RESEARCH**

**Query**

Fall Hospital

**Field**

Title

**Language**

English
